# Rationales and Approaches for Studying Metabolism in Eukaryotic Microalgae

**DOI:** 10.3390/metabo4020184

**Published:** 2014-04-11

**Authors:** Daniel Veyel, Alexander Erban, Ines Fehrle, Joachim Kopka, Michael Schroda

**Affiliations:** 1 Max Planck Institute of Molecular Plant Physiology, Am Muehlenberg 1, D-14476 Potsdam-Golm, Germany; E-Mails: veyel@mpimp-golm.mpg.de (D.V.); erban@mpimp-golm.mpg.de (A.E.); fehrle@mpimp-golm.mpg.de (I.F.); kopka@mpimp-golm.mpg.de (J.K.); 2 Molecular Biotechnology & Systems Biology, Technical University of Kaiserslautern, Paul-Ehrlich-Str. 23, D-67663 Kaiserslautern, Germany

**Keywords:** *Chlamydomonas reinhardtii*, metabolic engineering, biofuels, metabolite profiling, GC-MS, systems biology

## Abstract

The generation of efficient production strains is essential for the use of eukaryotic microalgae for biofuel production. Systems biology approaches including metabolite profiling on promising microalgal strains, will provide a better understanding of their metabolic networks, which is crucial for metabolic engineering efforts. *Chlamydomonas reinhardtii* represents a suited model system for this purpose. We give an overview to genetically amenable microalgal strains with the potential for biofuel production and provide a critical review of currently used protocols for metabolite profiling on *Chlamydomonas*. We provide our own experimental data to underpin the validity of the conclusions drawn.

## 1. Introduction

Fossil fuels are getting increasingly costly as their world-wide supply is declining, while global energy demands are steadily rising. Thus, extensive effort is currently being put into the development of renewable energy sources. In this context, microalgae receive significant attention as potential “biofuel crops” [1]. The intense interest in microalgae for renewable energy production has several reasons: algae are photosynthetic organisms that turn solar energy into chemical energy and other valuable products, thereby capturing CO_2_. Microalgae can be grown in open ponds or closed systems situated on non-arable land, and some species can even grow on waste or salt water, thus reducing fresh water consumption. The photosynthetic yield of microalgae is considered to be higher than that of conventional land-grown biofuel crops [1,2]. Microalgal diversity provides species able to produce molecular hydrogen, ethanol or triacylglycerides, which are easily accessible as biofuels [3,4]. However, the large diversity of microalgae has not yet been exploited for the production of biofuels, nor has the possibility of improving potential production strains by means of molecular breeding [1,2].

In this article, we give a short overview of microalgal strains amenable to metabolic engineering and of promise for biofuel production. We discuss the importance of systems biology approaches for the identification of targets for metabolic engineering and, in this context, focus on GC-MS-based metabolite profiling for the analysis of algal metabolism. With a focus on the model alga, *Chlamydomonas reinhardtii*, we review published methods for cell harvest, metabolite extraction and data normalization and support conclusions by our own experimental data.

## 2. Microalgae: Potential Candidate Species for Metabolic Engineering

The term “algae” denotes an artificial assemblage of diverse, photosynthetic eukaryotes that is polyphyletic and paraphyletic, including organisms that are evolutionary not of the same origin and excluding some of their relatives, such as land plants [5,6]. The prokaryotic, photosynthesizing cyanobacteria are sometimes also referred to as algae. According to their size, algae are classified as microalgae or macroalgae, with sizes of microalgae ranging from the 0.2–2 µm of single-celled picoplanktonic organisms up to 100 µm for filamentous algae. Algal habitats cover aquatic, subarial or benthic environments. Some aquatic algae are also tolerant of extreme environmental conditions of pH, temperature, salinity or heavy metal concentration [5,6].

Several microalgae are being used commercially in aquaculture for the production of carotenoids, polyunsaturated fatty acids, polysaccharides, animal feed and for health food [7]. Metabolic engineering has the potential to improve the quality and yield of these products and to generate strains capable of producing economically meaningful amounts of biofuels. As a basis for genetic engineering, many microalgal genomes have been sequenced recently, covering several taxonomic groups, like chlorophytes, eustigmatophytes, rhodophytes, coccolithophores, cryptomonads, prasinophytes, pelagophytes and diatoms. Many representatives of these taxonomic groups can be genetically manipulated and, therefore, are already amenable to metabolic engineering [8,9] (Table 1). Among these, we will highlight two species, *Nannochloropsis sp.* and *Chlamydomonas reinhardtii*; the former because of its high potential for genetic engineering and the latter because, on the molecular level, it is the best-characterized microalga.

*Nannochloropsis sp.* grows to high cell densities while tolerating a wide range of conditions with regard to pH, temperature and salinity. Upon nitrogen depletion, *Nannochloropsis* can accumulate oil exceeding 60% of its dry weight, and therefore, is an excellent candidate for biodiesel production [10]. The genome of *Nannochloropsis gaditana* has been sequenced recently [11] and transfection by electroporation or via *Agrobacterium* is established [11,12,13,14]. If the selectable marker harbors the 5′ promoter and 3′ UTR from different genes, integration into the genome occurs via nonhomologous recombination [14]. Given that vegetative *Nannochloropsis* cells are haploid, such constructs can be used for insertional mutagenesis in forward genetics screens. Most strikingly, transgenes flanked by sequences from the same locus also integrate into the nuclear genome via homologous recombination and, therefore, allow for targeted gene disruptions/replacements [11,14] (Table 1).

**Table 1:** Overview of genetically amenable microalgal species.

Species (group)	Transfection method ^a^	Transgene integration ^b^	Promoters ^c^	Selection marker ^d^	Reference
*Thalassiosira pseudonana* (Diatoms)	PB	NHR	*LHCF9* *NR*	*nat*	[15]
*Phaeodactylum tricornutum* (Diatoms)	PB	NHR	*fcpA*	*ble* *nat* *nptII* *sat-1* *cat*	[16,17,18,19]
*Nannochloropsis gaditana/oculata* (Eustigmatophytes)	EP AB	NHR HR Transient	*VCP1* *VCP2* *UEP* *βTUB* *HSP70* *HSP70-RBCS2*	*ble* *hygR* *bsr*	[11,12,13,14,20]
*Cyanidioschyzon merolae* (Rhodophytes)	EP PEG	HR Transient	*URA3* *Catalase* *βTUB*	*GFP* *URA3*	[21,22,23]
*Chlorella* (Chlorophytes)	PB PEG EP	HR Transient	*CaMV-35S* *Chlorella virus* *Chlamydomonas RBCS2*	*NR* *hpt* *nptII*	[24,25,26]
*Haematococcus pluvialis* (Chlorophytes)	PB EP	NHR Transient	*SV40* *Phytoene desaturase*	*Modified phytoene desaturase*	[27]
*Dunaliella salina* (Chlorophytes)	PB EP GB	NHR Transient	*Maize ubiquitin* *CaMV-35S* *Chlamydomonas* *RBCS2* *Actin* *CA* *NR*	*bar* *ble* *NR*	[28,29,30]
*Chlamydomonas reinhardtii* (Chlorophytes)	PB EP GB SCW AB	NH HR (chloroplast)	*HSP70A-RBCS2* *PSAD* *β_2_TUB* *NR* *CYC6*	*ARG7* *NR (nit1)* *ble* *aphVIII* *aph7“* *aadA*	[31,32,33,34,35,36,37,38,39,40,41]
*Volvox carteri* (Chlorophytes)	PB	NHR	*NR*	*NR*	[42]

^a^ PB, particle bombardment; EP, electroporation; PEG, incubation of protoplasts with polyethylene glycol; AB, *Agrobacterium*-mediated transfection; GB, agitation with glass beads; SCW, agitation with silicon carbide whiskers; ^b^ NHR, stable integration via nonhomologous recombination; HR, stable integration via homologous recombination; ^c^ LHC, light harvesting complex; NR, nitrate reductase; fcp, fucoxanthin chl a/c-binding protein; VCP, violaxanthin/chlorophyll a binding protein; TUB, tubulin; HSP, heat shock protein; UEP, ubiquitin extension protein; RBCS, Rubisco small subunit; URA, uracil; CaMV, cauliflower mosaic virus; SV, simian virus; CA, carbonic anhydrase; CYC, cytochrome c; ^d^ nat, nourseothricin resistance; ble, resistance to phleomycin antibiotics; nptII, kanamycin resistance; sat-1, streptothricin resistance; cat, chloramphenicol resistance; HygR, resistance to hygromycin B; Bsr, resistance to blasticidin S; hpt, hygromycin resistance; bar, resistance to herbicide phosphinothricin; URA3, complementation of ura3 mutant with wild-type URA3 gene; GFP, screening for cells expressing green fluorescent protein; NR, complementation of nitrate reductase mutant with wild-type NR gene; arg7, complementation of argininosuccinate lyase mutant with wild-type ARG7 gene; aphVIII, resistance to paromomycin, kanamycin and neomycin; aph7“, resistance to hygromycin B; aadA, resistance to spectinomycin and streptomycin; modified phytoene desaturase, norflurazon resistance.

*Chlamydomonas reinhardtii* is a unicellular green microalga living in freshwater environments. It is haploid during vegetative growth, and that can be interrupted by a sexual cycle, resulting in a single, diploid zygote that can resist adverse environmental conditions [43]. *Chlamydomonas* has emerged as a valuable model organism in research areas like photosynthesis and chloroplast biogenesis [44,45], the biology of flagella and basal bodies [46,47], cell-cell recognition [48] and circadian clock studies [49]. Moreover, *Chlamydomonas* has recently received much attention because of its elaborated fermentation pathways [50] and because of its ability to produce molecular hydrogen [51] and nonpolar lipids [3] that have promise as biofuels. With respect to lipids, *Chlamydomonas* starch accumulation mutants were shown to accumulate triacylglycerols to 46%–65% of dry weight under stress conditions, like nitrogen starvation [4]. 

All three *Chlamydomonas* genomes are sequenced (nuclear, chloroplast and mitochondrial) and all three genomes may be genetically manipulated [52]. A large number of molecular tools have been established for *Chlamydomonas*, including selectable marker genes [37,53], strong promoters [41,54], codon-adapted reporter genes [55,56], insertional mutagenesis methods [57], gene-targeting methods [58] and vectors to induce RNAi [59] or to express artificial micro-RNAs [35,60,61] (Table 1). The disadvantages of *Chlamydomonas* are inefficient homologous recombination and problems with the high-level expression of nuclear transgenes that are caused by efficient gene silencing mechanisms in this alga [38]. However, the available technical resources are by far the most advanced in *Chlamydomonas* when compared to other microalgae, and the knowledge gained from *Chlamydomonas* research can be transferred to promising, yet intractable, algal strains [2].

## 3. Systems Biology towards Microalgal Biotechnology

Efficient breeding by means of molecular biotechnology will be essential for the prompt economical applicability of microalgae for biofuel production [1,2]. Microalgae possess several favorable characteristics for rapid breeding, such as short generation times, easy mutagenesis and screening and rapid identification of interesting mutants in strains with a haploid vegetative growth phase [2]. However, a thorough understanding of metabolic pathways is mandatory for their successful engineering. Thus, the characterization of algal metabolism has become a key task in order to select and engineer algae for industrial use [2,62].

For several reasons, *Chlamydomonas reinhardtii* is an ideally suitable organism for systems biology: its unicellularity and ability to grow photo-, mixo- and hetero-trophically offers the potential to grow cultures under various and controlled environmental conditions [43]. Further, *Chlamydomonas* can be grown in continuous cell cultures that avoid the problems arising from tissue heterogeneity or developmental stages present in multicellular organisms. 

Systems biology approaches in general follow “bottom-up” or “top-down” approaches. In bottom-up approaches, the system may be a confined subprocess, like a regulatory circuit or a metabolic pathway, which may be iteratively modeled mathematically to generate hypotheses that are experimentally testable. By contrast, top-down approaches are characterized by a more unbiased attempt to grasp the complexity of a biological phenomenon (e.g. acclimation to an environmental change) as completely as possible with complementary high-throughput technologies. The generated comprehensive data may subsequently be used for correlative networks at different cellular levels in order to uncover underlying principles of regulation [63].

For systems biology approaches, metabolic network reconstructions provide a framework to characterize cellular metabolism and allow for the identification of potential bottlenecks and key enzymes in metabolic pathways, which are promising targets for genetic engineering [62,64]. Genome-scale models can be reconstructed based on the complement of metabolic enzymes using genome sequences, information on subcellular localization and thermodynamic and stoichiometric coefficients and, for this reason, are, in principle, also attainable for biochemically less well-studied organisms [65]. Once a stoichiometric model for an organism has been established and curated, *in silico* methods, like flux balance analysis (FBA), can be applied to predict metabolite fluxes under certain objective functions, such as maximum growth.

Genome-scale metabolic network reconstructions have been performed for *Chlamydomonas* only recently [62,66,67,68]. FBA was applied to the *Chlamydomonas* metabolic models for auto-, mixo- and hetero-trophic growth [67] or for H_2_-producing conditions [68]. In particular, the analysis of FBA outcomes suggested increased H_2_ production by strains with inhibited cyclic electron flow and decreased TCA cycle activity, metabolic properties that are indeed effective in the H_2_-producer mutant, *Stm6* [68,69]. In addition, this analysis could identify other potential targets for engineering to increase H_2_ production that deserve further experimental testing. Chang *et al.* (2011) included modes for different light source usage in their *Chlamydomonas* metabolic model to predict the most efficient light source in terms of absorbed photons and biomass yield [62]. The results showed that the use of red LED light (674 nm LED with a minimum incident photon flux of 362 µE/m^2^/s) was already very close to the theoretically most efficient light source (677 nm peak light spectrum with a total incident photon flux of 360 µE/m^2^/s) for *Chlamydomonas* growth [62] that not surprisingly coincides with maximum chlorophyll absorbance.

Finally, in addition to *in silico* predictions, the integration of multiple high throughput data sets into genome-scale models can lead to a refinement of the models that together can accelerate metabolic engineering processes [64]. However, successful prediction from metabolic models relies on precise parameters and, importantly, on a correct regulatory topology. Thus, the identification of unknown regulatory interactions represents a major future challenge for metabolic modeling and, hence, also for the metabolic engineering of microalgae. For a discussion of the current state on the modeling of metabolism, see [70].

## 4. Metabolite Profiling Using GC-MS

The measurement of metabolites in a systems biology experimental setup is considered as one of the most significant contributions to understanding cellular processes [71]. Among mRNA and protein abundances, metabolite pool sizes aid in characterizing the observed molecular phenotype, but only their integration allows a proper interpretation of regulatory processes [72,73,74].

Metabolites from biological materials are most frequently analyzed by chromatographic separation combined with mass spectrometry (MS) or by nuclear resonance spectroscopy (NMR) [75,76,77]. The separation of pre-fractionated cellular extracts using capillary electrophoresis (CE), gas chromatography (GC) or liquid chromatography (LC) and the subsequent analysis by different MS types simplifies the accurate identification and quantification of small molecules from complex samples. An unbiased measurement of cellular metabolites, however, is challenging, mainly due to their huge chemical complexity [73]. More comprehensive metabolite coverage is thus reached when several platforms are applied to one sample in parallel, because different separation (LC, GC, CE) and MS ionization (electron ionization, electrospray ionization, *etc.*) techniques favor compounds of different sizes and polarity [77,78]. This can result in the detection of several hundred compounds from a single sample [79]. Furthermore, specialized MS-based methods have been developed for the analysis of particular, but not habitually, accessible metabolites [80,81].

GC-MS is a method that was already used for metabolite profiling in the 1970s [82,83]. More recently, this technique has been recognized in plant research as a highly valuable metabolic phenotyping approach [71,84]. GC-MS, or more specifically, gas chromatography, connected to electron-ionization- (EI-) time-of-flight- (TOF-) MS, is a combination of two powerful analytical methods: GC separates volatile compounds based on their size and polarity with high capacity. Thereafter, EI-TOF-MS ensures reproducible analyte fragmentation and mass spectra acquisition at high scan rates [76]. However, many of the compounds in classical methanol-water soluble cell extracts have to be chemically modified to enhance volatility and stability before they can be separated by GC [78,85]. One of the most commonly used derivatization methods in plant metabolite profiling applications is the two-step derivatization, including oximation of keto-groups followed by trimethylsilylation of reactive functional groups [78,86]. GC-MS preferably detects less polar compounds, like hydrocarbons or fatty acid derivatives, and, due to the mass adducts produced by derivatization, smaller polar metabolites, such as sugars, sugar alcohols, amino acids, organic acids and polyamines [78,87].

The unambiguous identification of compounds measured with GC-EI-MS from complex biological extracts is only achieved by the combination of compound-specific retention time and mass spectrum, due to the existence of highly similar mass spectra retrieved from, e.g. conformational isomers [88,89]. The retention time of analytes can be standardized to units of highly reproducible retention indices (RI), such as the Kováts indices, by inclusion of retention time standards [87,88,90,91]. Publicly available retention-index/mass-spectral libraries as provided, e.g. by the Golm-Metabolome-Database [87,92,93], contain analyte-specific information on retention indices and mass spectra, which can be used for unambiguous annotation. In recent years, bioinformatics tools have been developed for the ease of handling, peak alignment and annotation and analysis of GC-MS-derived metabolite profiling data [94] (reviewed in Tohge and Fernie [95]).

## 5. From Experiment to Data: Metabolite Profiling Workflow for *Chlamydomonas*

**Figure 1:**
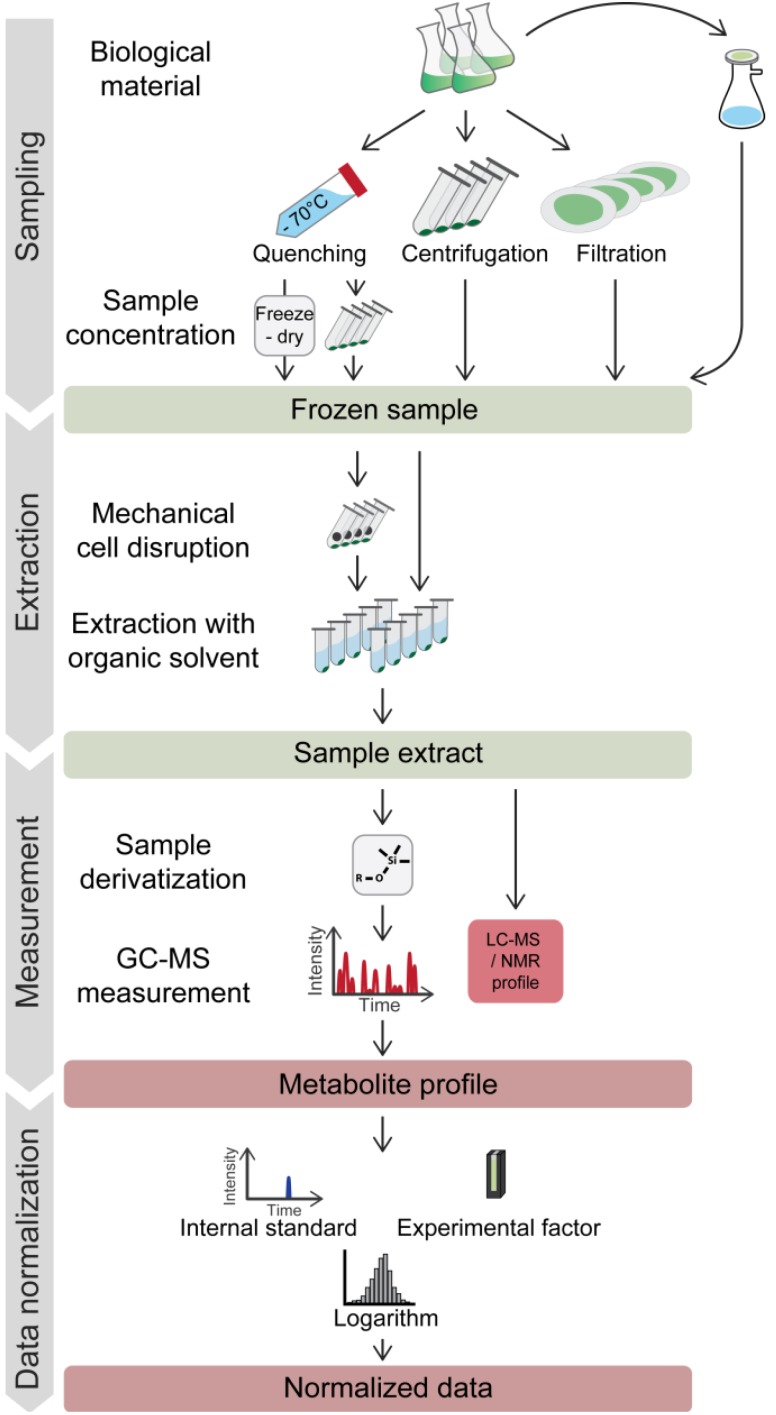
Typical workflow for metabolic profiling experiments on microorganisms. Harvesting of microbes for metabolite profiling may involve a rapid quenching step. Frozen samples are extracted with organic solvents, optionally including a grinding step. For GC-MS analysis, extracts have to be chemically derivatized to enhance the thermal stability and volatility of the compounds. Most frequently, the first standardization steps involve normalization to labeled internal standard(s) and to a reference value for the analyzed biomass (e.g. culture optical density (OD)) for the comparison of data from different samples. Logarithmic transformation is commonly utilized to achieve a near normal distribution of the data. For further descriptions, see the text.

Figure 1 shows a classical workflow of metabolite profiling on *Chlamydomonas* cells, including sampling, sample workup and, after GC-MS measurement, pre-treatment of the data. In the following section, we will discuss the important steps of this workflow and review the published protocols for *Chlamydomonas*.

### 5.1. Cell Harvest

The harvesting of biological material for the analysis of cellular metabolites is a challenging task. Sample handling during harvest will ultimately affect data quality and, hence, the conclusions drawn. This is particularly true for pools of metabolites with very short turnover times, like ATP/ADP or Calvin-Benson cycle intermediates, as was shown for *Arabidopsis thaliana* and *Chlamydomonas* [80,96]. Therefore, the harvesting method should arrest metabolism as quickly and entirely as possible (this process is often denoted as quenching) to ensure a true snapshot of the current metabolic state [97,98]. Quenching is normally achieved by an immediate decrease or increase in temperature or pH via subjecting the sample to suitable hot, cold, acidic or alkaline treatments [99].

Microalgal cultures are rather dilute (for instance, logarithmically growing *Chlamydomonas* cultures only contain up to 5 × 10^6^ cells/mL). Thus, ideally, the medium is removed during harvest to concentrate the cells and to remove medium salts that can interfere with analytical measurements. For example, remnants from *Chlamydomonas* growth media led to severe ion suppression in an LC-MS approach [96]. However, the process of separating cells from growth medium is time consuming and conflicts with a rapid arrest of metabolism. Therefore, the harvesting technique should be carefully adapted to the biological question asked and should be compatible with the following analytical platform. Major techniques used for the harvesting of microbes for metabolite analysis include methods that quench metabolism before (or without) the separation of cells from growth media and methods that quench after the separation of cells from growth media, like centrifugation or filtration.

#### 5.1.1. Quenching before (or without) Separating Cells from Growth Medium

In order to arrest metabolism immediately, the sampling of microorganisms for metabolite analysis is frequently done by injecting the sample instantly into liquids cooled to below −20°C, which contain a considerable fraction of organic solvent to maintain fluidity. This first quenching step is optionally followed by centrifugation to remove the medium and quenching solution. The most popular quenching method used to date is a methanol-water mix (60:40, v:v), cooled to −40 °C [100]. However, the high methanol content has been shown to result in the significant leakage of metabolites from bacterial, yeast and animal cells [101,102,103,104,105,106,107].

Due to the problem of metabolite leakage during quenching, alternative quenching solutions have been proposed for some prokaryotic and eukaryotic species, including pure methanol [106], pH buffered or isotonic methanol-water [108,109,110] and glycerol-water supplemented with sodium chloride [101]. However, despite these improvements, no universal quenching solution that completely prevents leakage was shown to be applicable to a wide range of species [104].

To date, two studies have investigated the effect of quenching on metabolite leakage in *Chlamydomonas*. Bölling and Fiehn [111] showed that metabolite leakage from *Chlamydomonas* cells was occurring after quenching in methanol-water (−20 °C, 32.5% methanol; final concentration of 24.4%), not surprisingly, leakage was found to be less from cells containing a cell wall than from cells lacking a cell wall. Using the walled strain, CC 125, quenching at lower temperatures and consequently higher methanol concentrations (−70 °C, 70% methanol; final concentration of 35%) was shown to reduce leakage for most metabolites to below 5% [112]. However, two other studies mentioned non-acceptable metabolite leakage following quenching in cold methanol-water using the wall-less strain, CC 503 (−20 °C; 100% methanol; final concentration of 30%; [113]), and the walled strain, CC 1690 (−70 °C; 70% methanol; final methanol concentration of 46.6%; [96]). This observation prompted these authors to measure cells with medium, thereby circumventing the metabolite leakage issue, while still benefiting from the rapid quenching of metabolism [96,113].

A more recent study investigated the metabolome of *Chlamydomonas* in response to CO_2_ limitation [114]. The authors used a cell wall-less strain with a quenching protocol adopted from Bölling and Fiehn [114]. Although quenching a wall-less strain in methanol-water is likely to result in extensive metabolite leakage [111,113], the authors of this study did not mention any controls or problems related to metabolite leakage.

#### 5.1.2. Centrifugation

Centrifugation is a common harvesting technique for microbes for all sorts of applications. However, due to the uncontrolled reaction of metabolism to darkness and centrifugal forces during centrifugation, this technique is not considered suitable for harvesting photosynthetic microbes for the analysis of metabolites. Two published studies used centrifugation to harvest *Chlamydomonas* cells during a time course of sulfur starvation to investigate the metabolome under H_2_ producing conditions [115,116] (Table 2). There was a remarkable difference in the amount of harvested cells between the two studies, yet the outcomes were comparable. For example, both studies revealed that the sulfur-starvation-induced anaerobiosis caused levels of TCA cycle intermediates to decrease and intermediates of glycolysis and the amino acids Ile, Leu and Tyr, to increase [115,116]. Hence, despite the problems mentioned, centrifugation in some cases might allow differences in levels of metabolites with large pool sizes and low turnover rates to be captured.

**Table 2:** Comparison of the methods for sampling, extraction and sample workup of GC-MS based metabolite profiling studies in *Chlamydomonas*.

Study	Strain used	Harvesting method ^a^	Harvesting conditions ^b^	Harvested cells ^c^	Mechanical cell disruption	Extraction buffer ^d^	Cells/mL extraction buffer	Extract equivalent to cells injected into GC-MS
[111]	CC 125	Q	32.5% MW; −25 °C; 4:1	×	mortar and pestle	MCW 10:3:1	1.20 × 10^6^	×
[112]	CC 125	Q	70% MW; −70 °C; 1:1	2.50 × 10^6^	5-mm steel ball	MCW 5:2:1	1.92 × 10^6^	1.68 × 10^4^
[113]	CC 503 cw92 mt+	Q broth	100% M; −20 °C; 0.43:1	×	none	MCW 1:1:0	×	×
[115]	Stm6	C	3,000 g; 1 min; 4 °C	3.00 × 10^7^	none	MCW 1:0:0	6.00 × 10^7^	1.00 × 10^7^
[116]	CC 406 & Stm6Glc4	C	3,000 g; 1 min	3.60 × 10^8^	homogenizer, 0.1-mm silica beads	MCW 4:0:1	3.60 × 10^8^	2.52 × 10^6^
[114]	cw 92	Q	32.5 MW; −25 °C; 4:1	1.50 × 10^6^	none	MCW 3:1:1	×	×
[117]	CC 503 cw92 mt+	Q broth	100% M; −20 °C; 1:1	6.00 × 10^6^	none	MCW 5:2:1	3.00 × 10^7^	5.31 × 10^4^
[118]	CC 125	Q	70% MW; −80 °C; 3:1	2.00 × 10^6^	sonicator (3 × 30 sec)	MCW 10:3:1	2.00 × 10^6^	2.00 × 10^4^
[119]	CC 125	Q	70% MW; −70 °C; 1:1	5.00 × 10^6^	5-mm steel ball	MCW 5:2:2	6.67 × 10^6^	8.97 × 10^4^
[120]	cw 15	F	×	3.50 × 10^7^	none	MCW 5:2:1	1.75 × 10^7^	1.75 × 10^5^
[121]	CC 503 cw92 mt+	×	×	15–25 mg fresh weight	Retsch mill, quartz sand	MCW 5:2:1 (1% acetic acid)	×	×
[122]	CC125	F	30–45 sec	2.00 × 10^7^	mortar and pestle	MCW 0:1:1	4.00 × 10^6^	1.92 × 10^5^
[123]	CC125	Q	70% MW; −70 °C; 1:1	7.00 × 10^6^	5-mm steel ball	MCW 5:2:2	9.33 × 10^6^	6.53 × 10^4^
This publication (see Experimental section)	CC 1690	F	10–20 sec	1.00 × 10^7^	none	MCW 7:3:0	1.39 × 10^7^	1.50 × 10^5^

× , no information given in the study. ^a^ *Q/C/F*, quenched/centrifuged/filtered; *Q broth*, quenched cells including medium. ^b ^The parameters used at harvest for a given method. For quenching, the composition of the quenching solution, its temperature and the quenching buffer-to-sample ratio are given; for centrifugation, speed, time and temperature are given; for fast filtration, the filtration time is given. ^c^ The lowest sampled amount indicated in the respective study. ^d^ *M/C/W*, methanol/chloroform/water.

#### 5.1.3. Fast Filtration

As a quick method for separating cells from the medium of microbial cell cultures, fast filtration represents an alternative harvesting method to quenching and centrifugation. The process from harvesting to freezing may be accomplished within around 10 sec [124,125]. Moreover, the filtration process may be done close to the respective experimental conditions (light, temperature, *etc.*), thereby keeping the disturbing influences of the sampling process at a minimum. Filtration of microbial cell cultures further provides the possibility to easily retain the filtrate for the analysis of secreted metabolites. Fast filtration was successfully employed to harvest various organisms for metabolite analysis, which showed strong metabolite leakage when quenched in cold methanol-water, including Gram-positive and Gram-negative bacteria [105,126,127,128], yeast [129], animal cells [107] and cyanobacteria [124].

Fast filtration of *Chlamydomonas* cells was already applied in the 1960s for the analysis of glycolate pathway metabolites [130]. Recently, a metabolomics study investigating the effect on NaCl-mediated abiotic stress in *Chlamydomonas* used fast filtration for harvesting. In this case, 5 mL of culture were filtered with 50-mm diameter filters [120]. Unfortunately, the study provided neither filtration times nor harvested cell numbers. In another study using NMR for metabolite analysis, 1 × 10^8^ *Chlamydomonas* cells were harvested per filter with diameters of 90 mm, which took between 30 to 45 sec [122]. 

Changes in cell size and shape influence the filtering speed and cell number capacity that can be captured on a filter. As the size of *Chlamydomonas* cells depends on environmental conditions, for instance in synchronized cultures [43], the amount of culture to be filtered needs to be adapted for each experiment to keep filtering times acceptably short. 

Other approaches than sampling microbes from liquid cultures for the analysis of metabolites have been demonstrated by transferring the cells (yeast or *E. coli*) from a liquid pre-culture to filter membranes briefly before the experiment. The filter discs with cells are then put on top of agar plates or constantly perfused with medium [98,131,132]. This setup was used to rapidly change the carbon source, for instance in the perfused medium, and allowed a fast quenching of the cellular metabolism by directly submerging the filter in hot extraction buffer [132]. However, these approaches may generate heterogeneity in the cells on the filter, as cells may be present in different layers that may suffer from shading or limited nutrient supply. Moreover, in cases where larger amounts of cells are required, scaling-up is difficult.

#### 5.1.4. Different Harvesting Methods Produce Distinct Metabolite Profiles of *Chlamydomonas* Cells

**Figure 2:**
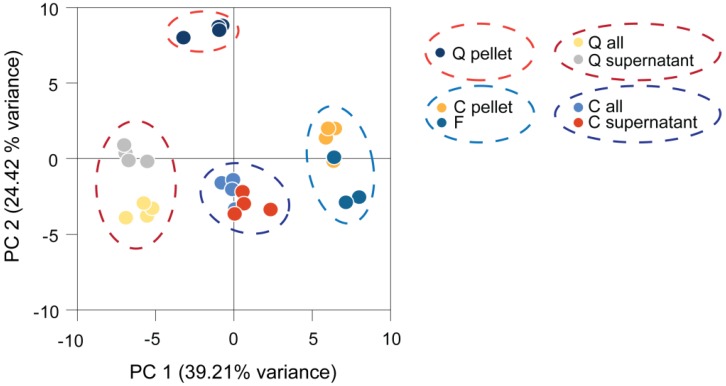
Principal component (PC) analysis of *Chlamydomonas* metabolite profiles obtained by different harvesting methods. Four replicates were harvested and processed from the same culture by each harvesting method and measured by GC-MS. The generated metabolite profiles were normalized to the internal standard and log_2_-transformed before being subjected to principal component analysis. C, centrifuged; F, filtered; Q, quenched; all, cells and medium.

We compared the harvesting methods discussed above for the analysis of *Chlamydomonas* metabolite profiles by collecting replicate samples from the same culture by quenching, centrifugation and fast filtration. Subsequent sample processing and metabolite analysis was identical. Some samples were analyzed together with the medium to reveal differences induced by the medium removal step. The resulting metabolite profiles were normalized to the internal standard, log_2_ transformed and subjected to principal component analysis (PCA). The results allowed the following conclusions (Figure 2). First, each harvesting method produced distinct, but consistent, data, as the different methods were separated while replicate samples clustered together. Second, four clusters of samples were separated by Principal Component (PC) 1 and 2: quenched samples containing medium, centrifuged samples containing medium, quenched samples only containing cells and centrifuged or filtered samples only containing cells. Profiles generated from filtered samples clustered with those from centrifuged samples, but were distinct from the quenched samples. Third, PCA clearly separated samples containing medium from the cells-only samples, while samples containing the supernatant without cells separated only slightly from the supernatant plus cells. This already indicates a minor contribution of the intracellular metabolites to these profiles (as further discussed below).

PC 1 separated mainly filtered and centrifuged samples from the medium-containing samples. The metabolites with the highest impact on PC 1 were found to be fatty acids, like linolenic acid, α-linolenic acid, palmitic acid, myristic acid and two unknown analytes (Golm Metabolome Database (GMD) identifiers A114002 and A361001), all of which had higher levels in centrifuged and filtered samples, while levels of erythronic acid, glycine and glycerol were decreased in these samples. PC 2 separation was mainly driven by high loadings of phytol, putrescine, glycerol-3-phospate, as well as iminodiacetic acid and an unknown analyte (GMD identifier A190021), resulting in differences of the quenched pellet sample from all other samples.

In summary, PCA demonstrates that each harvesting method seemed to produce distinct, but reproducible, data of an unperturbed *Chlamydomonas* culture, while centrifugation and filtration resulted in relatively similar metabolite profiles. The profiles of samples containing cells and medium together appeared to be majorly influenced by the medium part. Analogous results were obtained in a study of GC-MS-based metabolite profiling on cyanobacteria, where the same harvesting methods were compared [124]. Furthermore, in this study, consistent, but distinguishable, data were obtained by each harvesting method.

It has to be kept in mind that the data shown here were obtained by probing an unperturbed *Chlamydomonas* culture, thus underlining the reproducibility of each method. However, conditions inducing rapid changes in the levels of the analyzed metabolites might alter the outcome of this experiment. In this case, centrifugation likely would not be adequate to reliably capture these changes.

### 5.2. Metabolite Extraction

The ideal extraction method for metabolites acts as completely as possible and, at the same time, prevents the conversion or degradation of the extracted metabolites [133]. Most extraction methods exploit the effects of extreme pH, high temperature, organic solvents, mechanical stress or a combination thereof on the cell wall and cell membranes. Accordingly, metabolite extraction methods have been developed that utilize methanol or ethanol, hot or cold conditions and freeze/thaw cycles. A survey of extraction buffers revealed a trend towards more mild extraction solutions, like methanol and chloroform, in contrast to more harsh buffers containing strong acids or bases used in earlier times. This trend was driven by the need for extraction buffers that are compatible with today’s analytical methods, like LC- or GC-MS [133].

Buffers used for metabolite extraction from plant material frequently consist of methanol, chloroform and water (MCW) and are combined with milling [134]. While optimizing metabolite extraction from *Chlamydomonas* cells, Bölling and Fiehn (2005) obtained the best results with MCW at a ratio of 10:3:1 (v:v:v) [111]. When compared to the extraction buffer for *Arabidopsis*, the *Chlamydomonas* buffer contained a lower water content to account for water present in interstitial spaces in the cell pellet. More recently, [112] tested and compared the MCW (10:3:1) buffer from [111] with four different extraction buffers, including MCW (5:2:2), methanol-isopropanol-water (5:2:2), 100% methanol and acetonitrile-isopropanol-water (5:2:2) [112]. Overall, quantitative differences were found to be marginal between the different extraction buffers tested, most likely due to the high buffer-to-sample ratio. The authors finally concluded that MCW (5:2:2) performed best, because it provided the highest analytical precision. Extraction buffers containing chloroform were regarded as superior owing to the efficiency of chloroform to rapidly inactivate enzymatic activity during extraction [112].

As summarized in Table 2, the extraction buffers that were used for *Chlamydomonas* in different publications all consisted of very comparable solvent systems containing methanol, chloroform and water in combinations ranging from MCW 10:3:1 [111,135], MCW 5:2:2 [112,119,123], MCW 5:2:1 [96,120,121], MCW 3:1:1 [114], MCW 1:1:0 [113,136] to MCW 1:0:0 and MCW 4:0:1 [115,116] or MCW 0:1:1 [122].

The protocols mentioned above were mainly tailored for the extraction of polar and semi-polar metabolites for GC-MS analysis. However, including lipophilic metabolites in the analysis of cellular metabolites by LC-MS may provide valuable insights into another system level in top-down systems biology approaches. Especially under stress, the accumulation of TAGs in lipid bodies and the rearrangement and degradation of lipids represent major acclimation mechanisms (e.g. nitrogen or potassium starvation of *Chlamydomonas* or *Arabidopsis*) [4,137]. A universal extraction protocol for lipophilic, polar primary and secondary metabolites, as well as potentially proteins and starch from one sample may reduce the working time and technical variance. Such an integrative extraction protocol has been successfully applied to *Arabidopsis*, resulting in the detection of more than one thousand distinct compounds from a single sample [79]. For the extraction of polar and lipophilic compounds from a single sample, MTBE (methyl-tert-butyl ether) replacing chloroform in the classical MCW buffer has been shown to largely facilitate sample handling. After phase separation, the MTBE phase containing lipophilic metabolites ended above the aqueous phase due to its lower density, while starch and protein got pelleted [79].

Whether a mechanic disruption of *Chlamydomonas* cells is required for the efficient extraction of metabolites in the presence of organic solvents remains unclear. While studies designed to optimize the extraction protocol [111,112] did not test the extraction efficiency of extraction buffer alone, another study [113] mentioned that there were no differences between ground and non-ground samples. In fact, the tendency of extensive metabolite leakage especially from wall-less strains in methanol-water quenching solutions [96,111,112,113] suggests that metabolite extraction is efficient with the frequently used solvent systems of methanol, chloroform and water. To date, approximately half of the published protocols for metabolite extraction applied a mechanical disruption step, like sonication or grinding with a mortar and pestle, steel balls or quartz sand [111,112,119,120,121,122,135] (Table 2).

### 5.3. Sample Amounts, Matrix Effects and Extracellular Metabolites

Regarding the employed derivatization methods, machine settings and reference libraries, GC-MS is well established for plant metabolite profiling [77,84,86]. However, the quality of GC-MS metabolite measurements with respect to reproducibility and dynamic range may be affected by the complexity of the sample. In this context, the term “matrix effect” denotes all effects caused by constituents of an analytical sample affecting the quantitative result [138]. Matrix effects occurring in GC-MS measurements are often connected to limitations in derivatization capacity or efficiency in complex samples [139]. In addition, the analytical measurement can be affected by high sample complexity. In very complex samples, the probability of an analyte reacting with the surface of the GC is much smaller than in a less complex sample, which may, in turn, enhance the response of certain analytes in the complex sample [140]. Moreover, too high of a sample complexity can result in poor peak separation, as well as aberrant peaks or detector overloading, causing deconvolution problems and inaccurate quantification [138,141].

A comparison of published GC-MS protocols for *Chlamydomonas* revealed that the extract concentrations that were finally injected into the mass spectrometer differed considerably by two orders of magnitude (see Table 2). The above-mentioned problems with matrix effects are likely to occur at least for the highly abundant analytes when too concentrated extracts are measured. Therefore, the optimal amount of injected cells should carefully be determined to stay in the dynamic range of detection for most metabolites.

#### 5.3.1. The Linear Range of Biomass Concentration in Chlamydomonas Metabolite Extracts Is Limited

**Figure 3:**
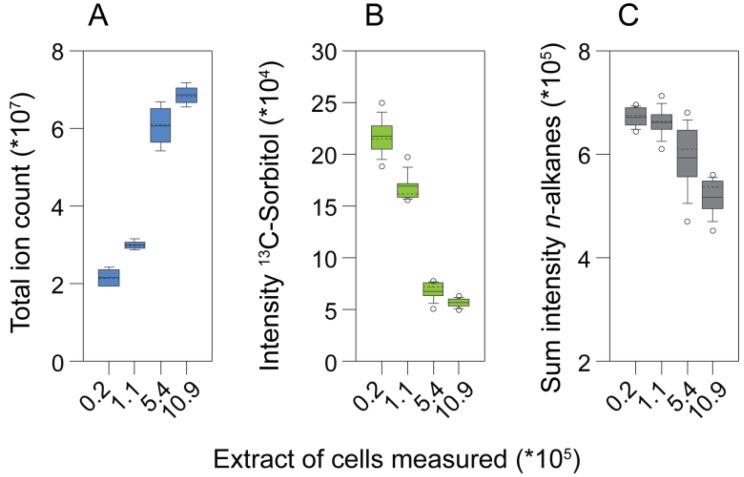
The effect of matrix complexity on metabolite profile properties. (**a**) Total ion count; (**b**) the response of the internal standard, ^13^C-sorbitol; and (**c**) the summed intensities of *n*-alkanes obtained by analyzing different amounts of cells.

**Figure 4:**
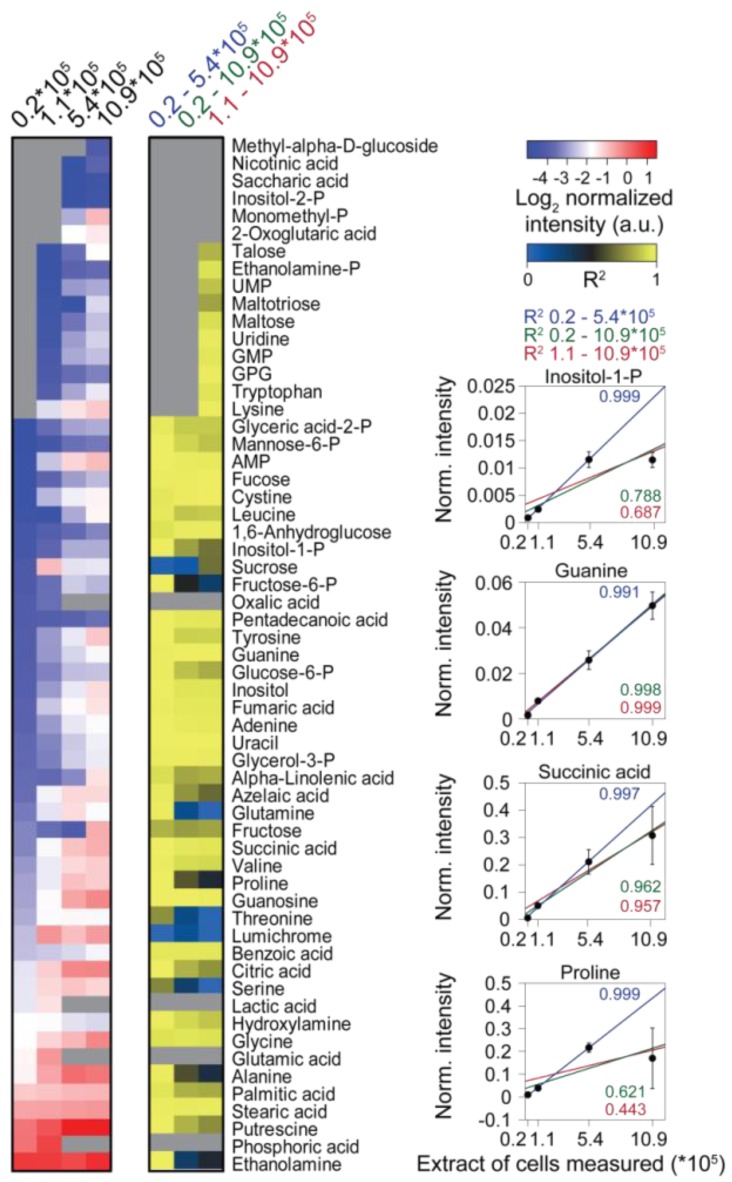
The effect of matrix complexity on the linearity of individual metabolite responses. (**Left**) Heat map of metabolites measured from differently concentrated extracts, the measured extract corresponding to the amount of cells is indicated at the top. (**Middle**) Heat map of the coefficients of determination (R^2^) values obtained by linear regression of the metabolite data from (left), including different samples as indicated at the top. Missing data are shown as grey boxes in the heat maps. (**Right**) The panel shows exemplarily the normalized abundance of four metabolites and the respective regression curves.

An experiment analyzing different extract concentrations with GC-MS revealed limitations when samples with high complexity were analyzed. The total ion count (TIC) of the chromatograms began to saturate when extracts equivalent to more than 5 × 10^5^ cells were injected into the GC-MS. By contrast, the intensity of the internal standard, ^13^C-sorbitol, already decreased between samples of 25 to five times lower extract concentration (Figure 3a). In addition, the responses of the retention time standards (*n*-alkanes), which do not get derivatized, decreased in the extracts of highest biomass concentration (Figure 3b). Consistently, the number of identified metabolites only increased marginally when extracts equivalent to more than 5.44 × 10^5^ cells were measured (Figure 3c; Figure 4, left heat map). Together these observations indicated that, already between samples with comparably low analyte concentration, quantitative performance may be disturbed, while in the samples with the highest analyte content, suppressing effects in GC-MS occurred.

Analysis of the responses of single metabolites showed good linearity for most metabolites in measurements of extract aliquots corresponding to up to 5.44 × 10^5^ cells. However, at higher extract concentrations, in particular, abundant metabolites had a dramatically disturbed linear response (Figure 3C). By contrast, less abundant metabolites, which could only be measured in extract aliquots equivalent to more than 1.1 × 10^5^ cells, showed good linearity in all samples detected. Strikingly, mainly analytes containing primary amines (like amino acids or putrescine) had a drastically disturbed linear response at higher extract concentrations, maybe owing to the limitations of the silylation reagent (*N*-methyl-*N*-(trimethylsilyl)-trifluoroacetamide (MSTFA)) in slowly reacting with primary amine groups [78,139].

A quite narrow linear range of analyte concentrations for GC-MS-based metabolomics has also been shown for extracts from adherent mammalian cells, for which the best linear range was obtained for extracts from 1.0 × 10^6^ to 7.5 × 10^6^ cells [142]. The authors showed that statistic comparison of differently concentrated extracts produced false significant results, i.e. significant differences between two differently concentrated extracts of the same culture despite normalization to the used biomass. These results are in line with our observation of the perturbed linear responses of certain metabolites when samples that are too concentrated are measured by GC-MS. In conclusion, the measurement of extracts equivalent to roughly 1–5 × 10^5 ^cells seems to provide reasonable coverage of the metabolites that can be identified, while still remaining in the linear range for most metabolites.

#### 5.3.2. Extracellular Metabolites and Growth Media Have Strong Impacts on the Sample Matrix in *Chlamydomonas* Metabolite Extracts

Growth media for *Chlamydomonas* contain high concentrations of TRIS, HEPES or phosphate as major buffer components [143]. These buffer substances are present at millimolar concentrations and thereby have a major effect on the sample matrix in metabolite extracts when samples with culture medium are used (Figure 2). Accordingly, matrix effects in GC-MS and LC-MS approaches were successfully diminished via adjusting the composition of *Chlamydomonas* growth media by changing the buffer system or by reducing excess nutrient components [96,113,114]. However, not only buffer and mineral salts in the growth medium can affect the sample matrix, but also secreted metabolites, like lumichrome, and fermentation products, like ethanol, lactate, glycerol, glycerate, *etc.* [130,135,144].

**Figure 5:**
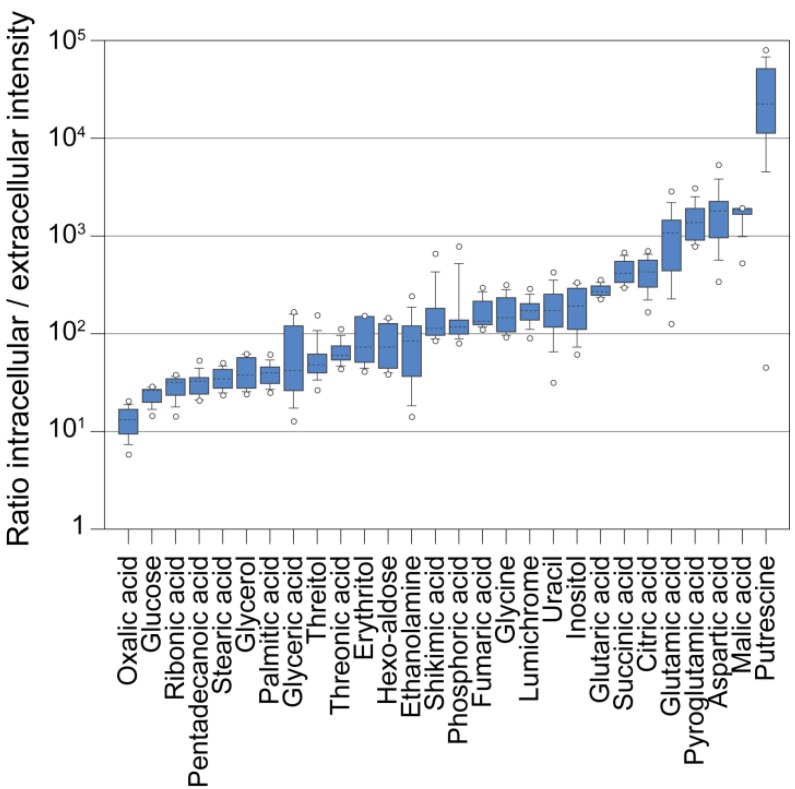
Ratios of intracellular to extracellular metabolite abundance. Measured metaboliteresponses that have been analyzed in both filtrate samples and cell samples were normalized to the extracted volumes (the total cellular volume was determined with a Coulter Counter), and the ratios of n = 36 samples were plotted as box plots.

In order to compare the levels of intracellular and extracellular metabolites, we grew a *Chlamydomonas* culture autotrophically in a semi-continuous bioreactor at low light intensity (200 µE/m^2^/sec) and at a constant optical density (OD) corresponding to approximately 3–4 × 10^6^ cells/mL. We harvested the cells by fast filtration and analyzed the cells and filtrate separately. Afterwards, we normalized the metabolite intensities to the corresponding volumes of cells and medium. Figure 5 illustrates that the intracellular concentration of virtually all measured metabolites was higher than it was in the medium (~10–1000 fold). However, the far excess of medium in a culture when measuring whole broth samples (2000 to 200 fold in a *Chlamydomonas* culture assuming 10^6^ to 10^7^ cells/mL, 10 µm cell size and spherical cell shapes) could cause a masking of the actual intracellular pool by extracellular metabolites (Figure 2) [104], besides strong matrix effects invalidating quantitative comparisons, as suggested by our experiments (Figure 3 and Figure 4). Moreover, in our experiment, we used a semi-continuous bioreactor that diluted the culture upon growth. In a batch culture, dead and lysed cells or higher cell concentrations may contribute even more to the extracellular complexity of metabolites. Following this, in order to get a true image of the intracellular metabolism of *Chlamydomonas* by GC-MS analysis, a removal of the growth medium during harvest seems essential.

### 5.4. GC-MS Data Normalization

A typical GC-MS-based metabolite profiling experiment consists of the comparative analysis of profiles from, e.g. different time points of a time series, different strains or wild-type *versus* mutant. The total variation observed in the experiment can be assigned to different sources, which are induced biological variance and uninduced variance, with the latter including uninduced biological variance and technical variance from sampling, sample workup and analytical measurement [145]. Normalizing metabolite profile data generally aims at reducing the uninduced variation without losing the intended biological variation and at preparing the data for subsequent statistical analyses.

In order to reduce technical variance, internal standards that are not present in biological samples are commonly added to the sample upon extraction [84]. However, in highly complex extracts with many compounds of various classes, concentrations of individual metabolites may be reduced as a consequence of tube wall adhesion, conversion or degradation. Thus, a single internal standard can trace such effects only to some extent. Hence, multiple internal standards or extracts of fully ^13^C-labeled cultures have been used to overcome these problems [98,146,147]. Another possibility to account for technical variability arising, e.g. from GC-MS machine sensitivity between samples is to normalize each metabolite to its median intensity measured during one day [84]. A different approach aiming at adjusting for technical signal drifts in large-scale experiments uses quality-control (QC) samples, which are run every fourth to fifth measurement and are used for fitting a LOESS (locally estimated scatterplot smoothing) regression curve [148] to each analyte feature. Subsequently, the analyte intensity in the experimental samples is corrected by interpolation of the regression curve [149]. This method, however, is relatively elaborate, due to the preparation of a universal QC sample containing all metabolites of interest, plus the frequent additional measurements of QC samples.

A typical source of unintended biological variation between samples derives from different amounts of harvested biomass. When working with plant organs or fruits, the data is frequently normalized to fresh or dry weight, because they are easy to determine [84]. The problem with microorganisms is that sample material is often limited, and it is, therefore, difficult to accurately determine the sample weight. Accordingly, alternatives that can be used are other measures of the harvested material (culture-specific properties) or factors intrinsic to the metabolite profile of the measured sample (profile-intrinsic properties) [150].

A suitable culture-specific property for single-celled microorganisms is the cell count, because the number of cells can be determined easily and fast. Cell count was used for normalization in a GC-MS-based study in *Chlamydomonas* [120]. However, results might get biased under conditions, leading to changes in cell size and, thus, sampled biomass. Moreover, accurate cell counting is impossible under conditions causing *Chlamydomonas* cells to become palmelloid. Another frequently used measure for microbe biomass is the optical density (OD) of the culture. OD600 is commonly used for many heterotrophic, homogeneously growing organisms [105,151]. For photosynthetic organisms, however, an OD at 750 nm has to be used to avoid chlorophyll absorbance [113]. Although the OD of a culture integrates cell count and size, it is also influenced by other properties of the cells, such as cellular granularity, which, in turn, depends on cellular constituents, like the cell wall or starch content [152]. Normalization may also be based on the harvested cell volume. Cell volume can be determined using a Coulter Counter^®^ under the assumption that cells have spherical shapes, albeit this might not always hold true for *Chlamydomonas* (e.g. when cells become palmelloid, after cell release in synchronized cultures or in some mutants [153]). Yet, compared with optical density, cell volume might be less influenced by cellular constituents. Finally, GC-MS metabolite profile data may be normalized to major cellular components, like starch, chlorophyll or total protein [150,154]. However, these factors again might be affected by the experimental condition. Moreover, if these measurements are of low accuracy, they will downgrade the results through the propagation of errors [150].

Apart from the mentioned culture-specific properties to account for differences in harvested biomass, chromatogram intrinsic properties can be used for this purpose. Profile-intrinsic properties, in contrast to culture-specific properties, have the characteristic to additionally correct for technical variation arising from, e.g. sample workup or machine sensitivity. Normalization to profile-intrinsic properties include the most frequently used total ion count (TIC) [84,142] or modifications of it. The TIC, regularly used for normalizing *Chlamydomonas* data [119,123], represents the sum of all measured metabolite abundances in one sample, while modifications of the TIC, like the median of the TIC or the TIC excluding the 5% most abundant metabolites, are more robust against extreme changes by a small set of metabolites [150]. Somewhat similar, the probabilistic quotient normalization (PQN) uses the median of the ratios of all individual features in the chromatogram to a reference chromatogram. The PQN was originally developed to normalize NMR metabolomic data [155], but was recently applied also to GC-MS data [122,154]. In addition, many methods that have actually been developed in recent years for standardization in microarray data analysis also are applicable for metabolomics data normalization [156,157]. Methods, like quantile normalization [158], variance stabilizing normalization [159] or cyclic locally weighted regression [148,160], have been compared on LC-MS and NMR metabolomics data [156,157] and have been used on GC-MS data [124].

After the successful reduction of technical and non-induced biological variance in GC-MS data, another important step of data pre-processing deals with the global data structure. GC-MS raw data often shows right-skewed distributions and is heteroscedastic, i.e. the data is not normally distributed, and the standard deviation of an analytical feature is proportional to its intensity [145,156,161]. A further property of metabolomics data is the potential huge difference in abundance between metabolites and the potential large fold change of the same metabolite between samples [145]. These properties are generally unfavorable for proper statistical evaluation of the data, and methods, like centering, scaling or transformation, are commonly applied for curing data from these factors [145,156].

Centering (subtraction of the mean over the samples from each respective metabolite value) positions the data around zero, while scaling alters data magnitudes and includes methods, like autoscaling (resulting in z-scores), range scaling, level scaling, *etc.* These treatments are mainly used to remove offsets in the data and to make each metabolite equally important [145]. Transformations, like the frequently applied logarithm or the square root (power transformation) are nonlinear conversions of the data. Transformations are generally used to make the data distribution (more) symmetric and are able to reduce heteroscedasticity [145,161]. The use of the most suitable transformation or scaling treatment depends on the data structure, data quality and biological question to be asked. They may have specific advantages and disadvantages, like suitability for biomarker identification or inflation of errors, *etc.* and, therefore, should be adapted to the individual experiment [145].

## 7. Experimental Section

### 7.1. Harvesting Methods Comparison Experiment (Figure 2)

For all experiments, *Chlamydomonas reinhardtii* strain CC 1690 was used. Cells were cultivated photoautotrophically in a semi-continuous bioreactor in H_5_AP medium [143,162] at a constant optical density corresponding to 3–4 × 10^6^ cells/mL at 200 µE/m^2^/sec. Quenching was performed by injection of 2 mL of culture in 2 mL of 100% methanol at −60 °C, centrifugation at 1,942 g, −10 °C for 5 min, separation of the supernatant and pellet and freezing in liquid nitrogen. For centrifugation, 2 mL of culture were centrifuged for 15 sec, 13,200 rpm at room temperature in a table-top centrifuge (Eppendorf AG, Hamburg, Germany). The supernatant was decanted, and samples were frozen in liquid nitrogen. Fast filtration was accomplished by filtering 2 mL of culture through polyvinylidene fluoride filters (47-mm diameter, 0.45-µm pore size, Merck Millipore, Merck KGaA, Darmstadt, Germany) with a suction flask connected to a vacuum pump (Vacuubrand GmbH + Co. KG, Wertheim, Germany). The filters were put in falcon tubes and frozen in liquid nitrogen. The whole filtration process took around 20 sec. All samples were stored at −80 °C until extraction. Extraction was started by adding 800 µL methanol:chloroform:water (MCW) (5:2:1, v:v) containing 4 µg/mL ^13^C-sorbitol cooled to −20 °C to the frozen sample followed by agitation for 5 min at 4 °C. A 5-min sonication bath treatment was followed by 1-h incubation on a rotor at 4 °C. After centrifugation for 10 min at maximum speed and 4 °C, 750 µL of the extract were vacuum-dried at room temperature and stored at −80 °C.

### 7.2. Extract Concentration Experiment (Figure 3 and Figure 4)

Cells were cultivated in flasks on a rotary shaker in TAP medium [143,162] at 35 µE/m^2^/sec and harvested at a cell density of 1.53 × 10^6^ cells/mL. Two, 10, 50 and 100 mL culture were harvested by centrifugation for 30 sec (SLA-3000, 4,500 rpm, Sorvall, Thermo Fischer Scientific, Waltham, MA, USA). The supernatant was discarded, and cells were resuspended in cold extraction buffer methanol: chloroform (MC) (2.33:1, v:v), containing 4 µg/mL ^13^C-sorbitol, transferred to a new tube and flash frozen in liquid nitrogen. After thawing, samples were incubated at 4 °C on a rotor for 1 h. To achieve phase partitioning, 400 µL of cold double-distilled water were added, and samples were centrifuged for 15 min, 13,200 rpm at 4 °C in a table-top centrifuge (Eppendorf AG, Hamburg, Germany). 700 µL of the upper, polar phase were vacuum-dried at room temperature and stored at −80 °C.

### 7.3. Extracellular Metabolite Experiment (Figure 5)

Cells were cultivated and harvested by fast filtration as described in the harvesting comparison experiment, except that 5 mL of culture were harvested per filter (filtration time ~30 sec). In parallel, 100 µL of the filtrate were collected and frozen. Cells were washed off from the filters in 3 consecutive steps of 600 µL of cold extraction buffer (MC (7:3, v:v), 2 µg/mL ^13^C-sorbitol, 20 µg/mL d4-alanine) and combined in a new 2-mL tube. After 1-h incubation on a rotor at 4°C, the extraction mixture was split into two fresh tubes, and 400 µL of double-distilled water were added to each sample. Phase partitioning was achieved by centrifugation for 10 min, 13,200 rpm at 4 °C in a table-top centrifuge (Eppendorf AG, Hamburg, Germany). 700 µL of the upper, polar phase were vacuum-dried at room temperature and stored at −80 °C. The filtrate samples were extracted identically to the filter samples, except that only 300 µL of double-distilled water were added before phase partitioning.

### 7.4. Metabolite Derivatization and Measurement

Metabolites were methoxyaminated and trimethylsilylated manually prior to GC-EI/TOF-MS analysis [71,84,88,93,163]. Retention indices were calibrated by the addition of a C_10_, C_12_, C_15_, C_18_, C_19_, C_22_, C_28_, C_32_ and C_36_ *n*-alkane mixture to each sample [91].

Metabolite profiling was performed as detailed previously [88,93] by gas chromatography coupled to electron impact ionization/time-of-flight mass spectrometry (GC-EI/TOF-MS) using an Agilent 6890N24 gas chromatograph (Agilent Technologies, Böblingen, Germany) with split and splitless injection onto a FactorFour VF-5ms capillary column, 30-m length, 0.25-mm inner diameter, 0.25-μm film thickness (Agilent Technologies, Böblingen, Germany), which was connected to a Pegasus III time-of-flight mass spectrometer (LECO Instrumente GmbH, Mönchengladbach, Germany). 

### 7.5. Data Pre-Processing and Peak Identification

GC-EI/TOF-MS chromatograms were acquired, visually controlled, baseline corrected and exported in NetCDF file format using ChromaTOF software (Version 4.22; LECO Instrumente GmbH, Mönchengladbach, Germany). GC-MS data processing into a standardized numerical data matrix and compound identification were performed using the TagFinder software [86,94,164]. Compounds were identified by mass spectral and retention time index matching to the reference collection of the Golm Metabolome Database (GMD, http://gmd.mpimp-golm.mpg.de/; [87,92,165]). Guidelines for manually supervised metabolite identification were the presence of at least 3 specific mass fragments per compound and a retention index deviation <1.0% [91]. Laboratory and reagent contaminations were evaluated by non-sample control experiments.

### 7.6. Data Processing and Visualization

Data processing, analysis and visualization were done with custom scripts in the *F#* programming language (Microsoft, Redmont, WA, USA). In detail, the maximum scaled numerical raw data matrix gained from the TagFinder software was imported into *F#* and normalized by the internal standard, ^13^C-sorbitol. Principal component and linear regression analysis were done on log_2_ transformed data by embedding the Accord.net framework [166]. Figures were drawn with the *F#* chart library [167] and reworked with Adobe Illustrator software (Adobe Systems, San José, CA, USA).

## 8. Conclusions

In this article, we have comprehensively reviewed the published protocols for GC-MS-based metabolite profiling on *Chlamydomonas*. Thereby, we focused, as far as accessible from the publications, on the employed harvesting methods of cells and extraction methods. In addition, we have presented data that encourage proper method establishment, and we have provided possibilities for normalizing GC-MS metabolite profiles from *Chlamydomonas* for subsequent reliable statistical analyses.

Up to now and despite a dozen publications on this topic, the harvesting of *Chlamydomonas* cells for metabolic profiling has not yet reached a universally applied standard. The quenching method in cold methanol-water represents the most frequently used technique (Table 2). While, apparently, quenching seems to be applicable for some relatively leakage-resistant strains, it is not recommended for the frequently used wall-less strains. In this respect, fast filtration represents the best alternative to quenching for GC-MS-based approaches. With harvesting times in the lower seconds range [124,126], fast filtration is superior to centrifugation, but has not yet been frequently used for metabolomics studies on *Chlamydomonas* (Table 2). There still seems to be room for harvest method improvements, such as combinations of quenching and filtration [168], different quenching solutions [169] or switching experimental setups, such as experimentation with cells on filters, which, so far, has only been applied to yeast and *E. coli* [98,132]. As suggested by our data, measurements of quenched whole culture samples (including growth medium) are only recommended for targeted approaches with adapted growth media, where the analyzed intracellular metabolites are not masked by extracellular excess and where matrix effects can be largely excluded [96,104]. The buffer systems used for *Chlamydomonas* metabolite extraction were found to be basically very similar and consisted of methanol, chloroform and water in different combinations (Table 2).

The analysis of different numbers of *Chlamydomonas* cells processed and measured by GC-MS revealed a limited linear range for biomass. This notion seems to be of importance for quantitative comparisons, because matrix effects in complex samples may strongly and differently affect the response of individual analytes. Therefore, biomass should be balanced between samples for crucial processing steps, like extraction, derivatization and GC-MS measurement. Hence, the amount of cells to be used is a trade-off: sufficient material to be above the detection limit for most metabolites, but as little material as possible to avoid inhibitory effects by the sample matrix. Our results suggest that extracts from 1–5 × 10^5 ^cells finally analyzed in the GC-MS seem to meet these requirements.

A thorough assessment of cellular biomass is necessary to reliably compare cellular metabolite levels between different samples, experiments or laboratories. The OD750 is an easy estimate for the biomass of *Chlamydomonas*; alternatively, the total cellular volume may be less influenced by the cellular composition. Other intracellular parameters, like total protein, starch or chlorophyll contents, could be used, but bear the risk of being affected by the biological treatment. Other methods to decrease inter-sample variance rely on the properties of each chromatogram, like TIC normalization and probabilistic quotient normalization (PQN). TIC normalization and PQN, in addition to methods derived from the standardization of microarrays, may thus be more robust against biologically-induced variation of culture-specific factors, like e.g. cell count. Yet, irrespective of the applied normalization technique, the suitable scaling or transformation represents an additional prerequisite to allow for the proper statistical analysis of GC-MS metabolomic data.

The use of systems biology to identify targets for the metabolic engineering of organisms is emerging [170,171] and attractive for the application to microalgae. Metabolomics and other high-throughput data can be used to screen microalgal strains and to refine or test predictions from genome-scale metabolic models [65]. Accordingly, GC-MS constitutes a valuable method for algal biofuel research. Nevertheless, to ensure that results are truly transferrable between experiments, organisms and laboratories, metabolomics methods, like GC-MS, should be reliably developed and should reach standards that go beyond the presently discussed issues [73].
